# Effect of Fertigated Water Consumption on the Immune Responsiveness and Antipredator Behavior of Red-Legged Partridge Chicks

**DOI:** 10.1021/acs.est.5c01484

**Published:** 2025-06-24

**Authors:** Elena Fernández-Vizcaíno, Mario Fernández-Tizón, Rocío Tarjuelo, Manuel E. Ortiz-Santaliestra, Rafael Mateo, François Mougeot

**Affiliations:** † 69555 Instituto de Investigación en Recursos Cinegéticos (IREC) CSIC-UCLM-JCCM,13005 Ciudad Real, Spain; ‡ Dpto. Biogeografía y Cambio Global, Museo Nacional de Ciencias Naturales (CSIC), 28006 Madrid, Spain; § Institute of Environmental Assessment and Water Research (IDAEA-CSIC), Jordi Girona 18, 08034 Barcelona, Spain

**Keywords:** Nitrate exposure, farmland birds, fertigation, antipredator behavior, immune responsiveness, physiological effects

## Abstract

Fertigation practices are common in dry agricultural areas, and nitrate exposure through fertigation water consumption could pose significant risks to farmland birds. This study simulated a realistic exposure scenario to evaluate the effects of drinking nitrate-contaminated water on the growth, physiology, and antipredator behavior of red-legged partridge (*Alectoris rufa*) chicks. Hatchlings were exposed to two nitrate concentrations commonly used in fertigation (100 and 500 mg/L) through *ad libitum* drinking water over a 28-day period. Nitrate exposure at both concentrations increased chick weight and elevated hematocrit levels, possibly as a compensatory response to nitrate-induced methemoglobinemia. Additionally, it reduced cell-mediated immune responsiveness, indicating a compromised immune function. We simulated aerial and terrestrial predator attacks (raptor and fox) and evaluated behavioral responses of exposed and control chicks. We report nitrate effects on antipredator responses, specifically reduced fleeing distances, altered freezing, and active escape behaviors, which would increase predation risk in the wild. These findings highlight new threats associated with nitrate contamination in agricultural landscapes, particularly for exposed birds that rely on irrigation water during the summer months. This study emphasizes the need to assess the sublethal effects of nitrates in high-exposure scenarios to improve environmental risk assessments and mitigate risks contributing to the population declines in farmland species.

## Graphical abstract

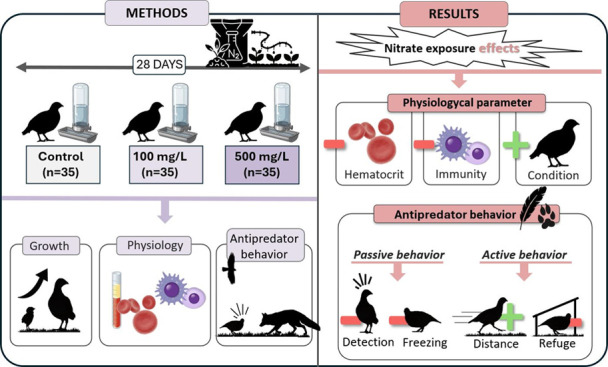

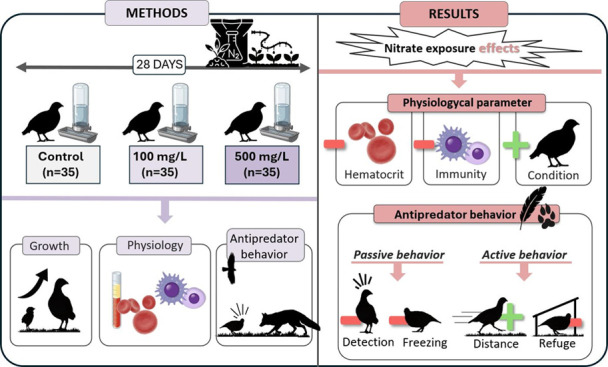

## 1 Introduction

Agricultural intensification is a main cause of farmland bird declines.^1,2^ One practice derived from intensification is the use of nitrogenous fertilizers, which has doubled since the 1940s^3^ and is continuously increasing worldwide.^4^ Many agricultural areas, especially their aquifers, are heavily contaminated due to excessive use of chemical fertilizers.^5,6^ Although nitrogenous fertilizers promote rapid crop growth, they also stimulate nontarget plant species, disrupting the nitrogen balance in the ecosystem and shifting the growth-limiting factor to other nutrients.^7^ In Europe, vulnerable areas to nitrogen compound contamination are those where nitrate levels exceed 50 mg/L^8^ because nitrate concentrations above 44.3 mg/L in drinking water are considered unsafe for humans. An excess of nitrate in water has been shown to have negative effects on domestic and wild animals^9,10^ and can significantly impact biodiversity.^11,12^ In Spain, concentrations over 100 mg/L nitrates have been found in the aquifers of intensive agriculture areas.^13–11,129,10^ To mitigate the excessive use of nitrogen in agriculture, it is common to add these fertilizers directly to irrigation water, a process known as fertigation. This practice helps reduce the total amount of nitrogen released into the environment and minimizes the risk of nitrate contamination in groundwater and surface waters,^16^ making it especially recommended in areas vulnerable to agricultural nitrate pollution.^17,18^ However, there is a significant risk associated with the direct consumption of fertigation water by birds, especially during the summer months in semiarid areas like Spain when there is a scarcity of water and a greater use of irrigation. This constitutes a potentially significant exposure route to high nitrate doses, since the nitrate concentrations recommended for fertigation often exceed safe levels, reaching values well above 100 mg/L.^19^

Nitrate (NO_3_^–^) toxicity in vertebrates has been linked to methemoglobinemia,^20^ caused by the oxidation of hemoglobin to methemoglobin by the nitrite (NO_2_^–^) primarily formed via bacterial nitrate reduction in the digestive tract.^21^ This impairs hemoglobin’s capacity to bind and transport oxygen,^22–24^ which could lead to oxygen insufficiency in different tissues, causing generalized tissue hypoxia that can ultimately lead to death.^25^ In the digestive tract, bacteria can also reduce nitrate to ammonia (NH_3_), which could disrupt normal nervous system function in animals by altering glutamate neurotransmission.^26,27^ These properties of nitrates, demonstrated at the molecular level, can result in a range of effects in birds such as delayed growth, reduced immune responses, reproductive failures, behavioral alterations, or death.^28–33^ Adult red-legged partridges (*Alectoris rufa*), exposed to nitrate concentrations commonly found in fertigation water experienced methemoglobinemia, lymphocyte DNA damage, increased oxidative stress and exhibited adverse effects such as reduced immune response and impaired growth.^31^ However, there is still a lack of studies evaluating nitrate toxicity effects at higher levels of biological organization in wild birds, specifically considering environmentally relevant exposure routes, as most studies assessing nitrate exposure in birds have focused on dietary additives in poultry to enhance productivity.^34–36^ In contrast, investigations into more complex end points, such as behavioral alterations, have been mainly conducted on aquatic organisms, which are more directly exposed to waterborne contaminants such as nitrates.^37–39^ For example, nitrates have been found to interfere with sensory processes and disrupt predator–prey behavioral interactions in amphibians.^39,40^ Studies investigating the effects of nitrite exposure on antipredator behavior in farmland birds are currently lacking, although alterations in predatory threats following sublethal exposure to other agrochemicals have been documented.^41^

Farmland birds, such as the red-legged partridge, inhabit agricultural areas and are therefore at a high risk of nitrate exposure. Moreover, irrigated crops, such as vineyards, provide a crucial water source for partridges during summer,^42^ affecting their local distribution.^43^ Many regions in central Spain are classified as vulnerable to nitrate pollution, where the practice of fertigation is widely used and irrigation aquifers have been found to contain nitrate levels exceeding 100 mg/L.^13,44^ These regions host important populations of red-legged partridges, a farmland bird of high socioeconomic importance in Spain that has experienced recent population declines^45^ linked to agricultural intensification^46^ and the use of agrochemicals.^47–51^ This species, which is common in the semiarid Mediterranean farmlands of the Iberian Peninsula, is particularly well-suited for evaluating the risks associated with drinking contaminated water.^52^

Exposure of partridges to fertigated water could result in deleterious effects on individuals due to nitrate toxicity. This risk is particularly relevant for partridge chicks, as their development occurs during the summer months,^53^ when irrigation use is at its peak and water resources are scarcer, increasing the likelihood of using nitrate-contaminated water sources. Despite this high risk, literature evaluating the impact of nitrate exposure through environmentally relevant pathways, such as fertigation, is limited in wild birds. Therefore, to adequately characterize this risk, experimental studies that assess effects are needed for extrapolating the results to real-life scenarios. In this context, we designed the present experiment simulating a realistic exposure scenario to investigate the effects of nitrates dissolved in drinking water (using concentrations found in fertigation water) on hematological parameters, cell-mediated immune responsiveness, and chick growth. We also tested for the first time effects on antipredator behaviors of red-legged partridge chicks to assess the effects of nitrate exposure at more complex levels of biological organization, such as prey–predator interactions. We hypothesized that nitrate exposure through drinking water, depending on the dose, would have adverse effects on the growth, physiology (reduced immune responsiveness), and antipredator behavior of red-legged partridge chicks.

## 2 Materials and Methods

### 2.1 Experimental Design

We conducted the study at the experimental farm “El Chaparrillo” Agro-Environmental Research Centre located in Ciudad Real Province, Castilla-La Mancha, Spain. Castilla-La Mancha harbors one of the largest populations of red-legged partridges that has dramatically declined in recent decades.^46^ Moreover, 66% of the region’s agricultural surface was recognized as particularly vulnerable to nitrate pollution.^54^ All procedures for the experiment were approved and authorized by the regional government of Castilla-La Mancha (permit ref. 9-2019). The experiment began on June 3, 2019, when red-legged partridge chicks were in their developmental stage (June–August^53^), coinciding with the peak of crop irrigation due to the dry summer and limited water resources. We previously collected eggs laid by captive partridge pairs breeding at the experimental center and incubated them for 21 days at 37.7 °C and 45% humidity and with constant movement. Eggs were then transferred to a hatching chamber at 37.7 °C with 60% humidity and without movement. Once the chicks hatched, they were individually tagged (with a leg band) and randomly assigned to one of three treatments. We used a total of 105 seven-day-old chicks, which were sexed a posteriori using a blood sample and a molecular sexing test (following Griffiths et al.^55^).

The experiment involved 35 partridge chicks in each treatment group, with an exposure period lasting one month, beginning 7 days after hatching. One group served as the control and was provided with commercial mineral water without nitrate throughout the experiment. The other two treatment groups were provided with the same commercial mineral water with the addition of a given sodium nitrate (NaNO_3_; Sigma-Aldrich) concentration. We selected these nitrate concentrations based on a previous work^31^ and using the levels found in nitrate-contaminated aquifers in some areas of central Spain (Castilla La Mancha; >100 mg/L),^13,31^ which are commonly used for irrigation and a key water source for wild birds during the summer. Additionally, we considered the nitrate concentrations recommended for fertigation practices in Spain (100–300 mg/L),^19^ which, when combined with the existing nitrate levels in irrigation water, could lead to significantly higher exposures, taking into account that in some cases both concentrations can be summed in agricultural scenarios. Moreover, field data showed that nitrate levels can be occasionally as high as 1000 mg/L in fertigated intensive vineyards of central Spain (authors, unpubl. data). This approach ensures that our study reflects realistic environmental scenarios in which wild birds may be repeatedly exposed to the nitrate concentrations used in the experiment.

Drinking water in the low-dose treatment group was prepared with a nitrate (NO_3_^–^) concentration of 100 mg/L (in the form of sodium nitrate; hereafter, referred to as N100). In the case of the high-dose treatment group, drinking water was prepared with a nitrate concentration of 500 mg/L in the form of sodium nitrate (hereafter, N500). Experimental drinking water dilutions were prepared by diluting the corresponding volumes of a stock solution elaborated from the dilution of the sodium nitrate salt in Milli-Q water. To minimize deviations from nitrate nominal concentrations, we prepared a new stock of diluted treated water approximately every 5 days, replacing the existing water a total of 5 times throughout the exposure period. We characterized nitrate exposure by measuring the initial and remaining water volumes to estimate average water consumption in each treatment group, assuming minimal evaporation from the drinking water containers. Each batch of chicks was housed in a heated closed room (2 × 2 × 2 m) with free access to an outdoor area (2 × 4 × 2 m). Throughout the exposure period, chicks were provided *ad libitum* food (initiation partridge chick fodder; Nanta-Nutreco, Tres Cantos, Spain) and water (treated or untreated). We ended the exposure period when chicks were 35 days of age (i.e., exposure lasted 28 days) and provided all groups with untreated mineral water afterward. Using the estimated daily water consumption per individual, the final body weights (b.w.) of partridges at the end of the exposure, and the theoretical nitrate concentrations for each treatment, we calculated a daily dietary dose (DDD) of nitrate (mg NO_3_^–^/kg b.w./day) during the exposure period for each treatment group. Birds were maintained in the same facilities until 120 days after exposure when the experiment concluded.

### 2.2 Chick Growth, Conditions, and Physiological Parameters

To study the effects on chick growth and body condition, we measured weight, wing length, and tarsus length at three time points: at the beginning of the experiment (7 days of age), at the end of the exposure period (35 days of age), and one month later (60 days of age). The body condition index for each time point was calculated as the weight corrected for tarsus length, following Peig and Green.^56^ After the exposure period, we extracted blood from each chick via a jugular venipuncture. Blood samples were kept refrigerated in heparinized tubes until they were processed in the lab. The percentage of hematocrit was measured using a 10 μL glass capillary tube, which was centrifuged at 10,000 rcf for 10 min to separate the plasma from the cellular fraction.

### 2.3 Cell-Mediated Immune Responsiveness

We measured cell-mediated immune (CMI) responsiveness in red-legged partridge chicks using the phytohemeaglutinin (PHA) skin test, as in previous studies on this species.^57,58^ The test involves an intradermal PHA injection that stimulates a perivascular accumulation of T-lymphocytes followed by macrophage infiltration.^59^ Prior to injection, we measured the thickness of the wing web at a premarked site using a pressure-sensitive dial thickness gauge (Mitutoyo Absolute 547X315) with 0.01 mm precision. Between 9:00 and 11:00 AM, each chick was injected with 0.5 mg of PHA (SIGMA L-8754) in 0.1 mL of physiological saline solution (PBS) at the marked location. Wing web thickness was remeasured 24 h postinjection, and the swelling response was calculated as the change in average thickness. This swelling reflects the first phase of the CMI response, corresponding to T-cell infiltration.^60^

### 2.4 Antipredator Behavior Test

At the end of the exposure period, we evaluated the response of 35-day-old chicks to a simulated predator attack. We designed an outdoor predator exposure system to recreate a realistic raptor or fox approach (see diagram, Figure S1a). Each chick was placed individually in a 50 × 50 × 50 cm cage made of a transparent metal mesh. This cage (initial position) was connected by a sliding door to a 6-meter-long corridor (50 × 50 cm) with a covered refuge at the end. After a 5 min acclimation period in the initial position, the door of the cage was opened by pulling a string, minimizing human interaction, and allowing access to the escape corridor and refuge (Figure S1a,b). Once the door was open, we simulated a predator approach (raptor or fox), which was hidden from the chick at the start of the test. The aerial predator (raptor) attack was simulated by presenting the wooden-plank silhouette of a medium-sized bird of prey (painted as a harrier) attached to an automatic pulley system (Figure S1c). The raptor silhouette descended from 6 m to just above the cage where the chick was placed at the onset of the test at a speed of approximately 0.5 m/s. The terrestrial predator (fox) attack was simulated by pulling a stuffed red fox placed on a cart that moved over ground rails and was pulled by a hidden operator using a transparent thread (Figure S1d). The fox decoys started 6 m away from the cage, approaching the test cage of the chick at a speed of approximately 0.2 m/s.

Observers recorded the behavioral responses of the chicks from a hideout placed at 4 m without knowing the treatment assigned to each chick. Each chick was tested twice in succession (with the raptor and the fox decoys, varying the order of predator presentation, raptor vs fox first, between chicks). Before the simulated attack (acclimation period), we recorded the bird’s state (nervous vs calm). During the simulated predator approach, we recorded the following responses: fleeing distance (i.e., the distance, in m, to the predator when the partridge reacted), alarm calling (yes vs no), freezing behavior (freeze vs no freeze), maximum escape distance within the corridor (displacement from 1 to 6 m), and refuge use (whether the chick reached the refuge to hide or not).

### 2.5 Statistical Analysis

Details on the statistical analysis are provided in the Supporting Information. We used the R software (R Core Team, 2020) for statistical analyses. Differences between treatment groups were tested using General Linear Models (GLMs) with normal (e.g., wing, weight, condition, hematocrit, CMI, escape distance) or binomial (probability of calling, freezing, or reaching the refuge) distributions. Initially, models included all factors, covariates, and their two-way interactions. Nonsignificant terms were subsequently eliminated (backward selection) starting with interactions. The treatment factor was retained in the models to report on the significance of treatment effects. Tukey tests were used to test for pairwise differences between different treatment groups. For all tests, the significance level was set at *p* < 0.05. *p*-values between 0.05 and 0.1 were reported as marginally significant effects.

## 3 Results and Discussion

Our experiment showed that exposure of partridge chicks to nitrate concentrations in drinking water commonly used for fertigation in agricultural areas altered physiology and behavior. We found increased hematocrit levels and reduced cellular immune responsiveness, compromising the immune status of chicks exposed to nitrates along with altered behavioral responses to simulated predation attacks. We found no direct lethal effects on exposed chicks, but the reported sublethal effects could increase susceptibility to parasites or diseases and reduce their ability to escape predators, potentially affecting individual survival and compromising population viability in intensively farmed environments. Based on the average water consumption per treatment (mean of 0.023 ± 0.007 L/day; Table S1) and body weight of each chick (mean of 0.151 ± 0.02 kg recorded at the end of treatment; Table S1), we obtained DDD values of 0, 15.50 ± 5.32, and 75.22 ± 19.47 mg of NO_3_^–^/kg b.w./day for the control, low, and high treatment groups, respectively.

### 3.1 Sublethal Effects in Bird Physiology and Condition

We observed significant effects of nitrate-fertilized water exposure on hematocrit levels (*Χ* ^2^ = 35.00, 2 d.f., *p* < 0.001; Figure 1a), which were 10% and 14% higher in the N100 (*p* < 0.001) and N500 treatment groups (*p* < 0.001) than in the control group, respectively. This response can be interpreted as a compensatory mechanism for a well-documented adverse effect of nitrates: methemoglobinemia.^20^ When nitrate is reduced to nitrite by digestive bacteria and enters the bloodstream, it oxidizes hemoglobin to methemoglobin, which cannot efficiently transport oxygen,^21,61^ leading to hypoxic conditions that can cause tissue damage or even death.^23,25,62,63^ Under such hypoxic conditions, the organism may compensate by increasing erythrocyte production to improve oxygen transport,^64^ which would explain the elevated hematocrit values found in our study. Increased hematocrit has been previously reported in birds after nitrate exposure.^65^ Atef et al.^66^ found that fowls (*Gallus gallus*) fed a diet with 4.2 g of nitrate per kg of food initially showed an increase in erythrocyte numbers, followed by a significant decrease, with methemoglobinemia present throughout the exposure. However, methemoglobinemia is not always associated with an increase in the hematocrit. In a similar study, Rodríguez-Estival et al.^31^ found elevated methemoglobin levels in partridges exposed to 100 mg/L nitrate for 14 days without any changes in hematocrit. Likewise, Strnad and Persín^67^ reported increased methemoglobin in pheasant chicks exposed to 500 mg/L nitrate, and González Delgado et al.^68^ observed higher methemoglobin levels in rats after oral subacute exposure of 13.86 to 109.41 mg/kg nitrate for 10 days; neither of them found changes in hematocrit. These inconsistencies in hematocrit results could be due to differences in exposure periods, dosage, species or age of exposed organisms.^69^ Furthermore, some studies have shown a positive time- and dose-dependent relationship between nitrate exposure and methemoglobin levels, suggesting that compensatory mechanisms may decline over time due to resource exhaustion or hemolytic anemia due to increased fragility of erythrocytes by oxidation properties of nitrates.^63,66^ The increased hematocrit observed in nitrate-exposed chicks could also be indicative of dehydration, a common physiological response in birds associated with hypoxia.^69^ In fact, the presence of nitrate in drinking water marginally increased water consumption in a previous study performed with adult red-legged partridges.^31^ In our study, however, water consumption increased over time (*Χ* ^2^ = 6.41, 1 d.f., *p* = 0.011) but did not significantly differ between treatment groups (*Χ* ^2^ = 0.56, 2 d.f., *p* = 0.775). Nevertheless, this potential sign of dehydration could have significant implications in natural environments, especially during summer when water sources are limited, and birds could consume more contaminated water to compensate for dehydration, thereby reinforcing their exposure to nitrates.^31^

**Figure 1:**
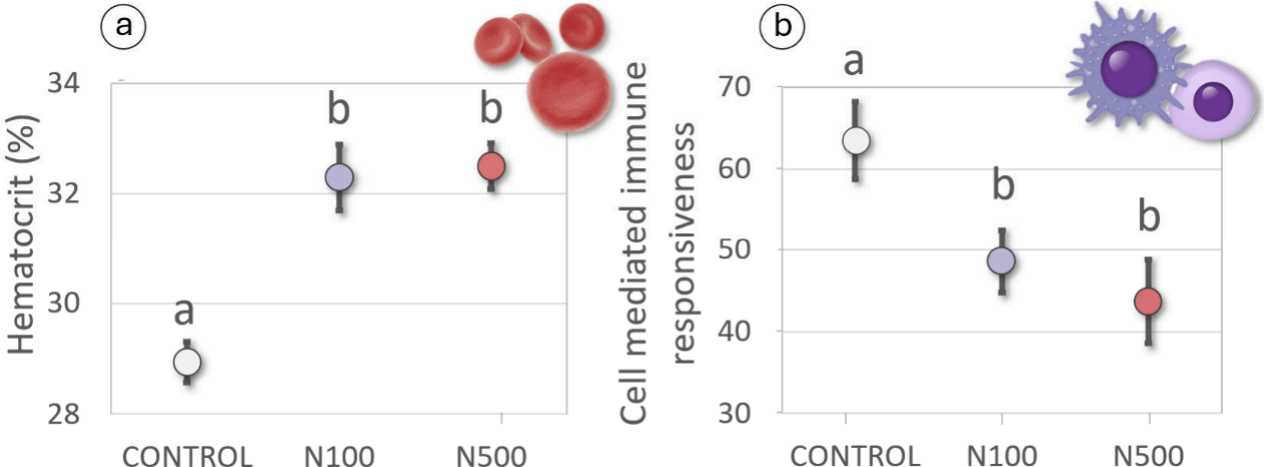
Treatment effects on physiology. Mean (±SE) hematocrit levels (a) and cell mediated immune responsiveness (b) of partridge chicks according to treatment. Within each graph, different letters above error bars indicate significant differences between groups (*p* < 0.05).

We detected significant effects of nitrate-fertilized water on the immune responsiveness of exposed partridges (*Χ* ^2^ = 9.89, 2 d.f., *p* = 0.007; Figure 1b). CMI responses were 21% and 31% lower in the N100 and N500 treatment groups compared to the control group, respectively, these differences being significant (*p* = 0.026; *p* = 0.003, respectively). These results indicate that nitrate exposure had a suppressive effect on T-cell-mediated immunity in exposed chicks, suggesting a decrease in T-lymphocyte and macrophage infiltrations.^59,70^ A similar effect was observed by Rodríguez et al.^31^ in adult partridges exposed to the same concentrations used in our study where exposed birds had reduced T-cell-mediated immune responsiveness and evidence of oxidative DNA damage in lymphocytes. In fowls, nitrate and nitrite impair humoral and cell-mediated immune responses by reducing leukocyte numbers, phagocytic activity, and overall immune function.^65,66^ As has been previously reported in birds exposed to the same doses of nitrate,^31^ we did not find statistical evidence for dose-dependent effects, possibly because these may occur at lower doses (between 0 and 100 mg/L).

The reduction in cellular immune response observed in our study, along with previous findings, could be due to the oxidative properties of nitrite,^71^ which can damage circulating white blood cells and hematopoietic tissues, leading to a decreased production of new leukocytes.^72^ Since the immune system is crucial for defending against pathogens and maintaining health,^73,74^ a weakened immune state raises the risk of infections and diseases in wild birds exposed to nitrates. Parasites and other pathogens can limit or regulate wild red-legged partridge populations,^75^ particularly affecting breeding females in heavily managed agricultural estates,^76^ and these effects may be exacerbated by exposure to nitrates or other contaminants.

Treatment had no apparent effect on chick growth. We found no effect on wing or tarsus length at the end of the exposure period (35 day-olds), only differences between sexes (males are larger than females; wing: *Χ* ^2^ = 14.68, 1 d.f., *p* < 0.001; tarsus: *Χ* ^2^ = 22.02, 1 d.f., *p* < 0.001). When analyzing body condition (weight corrected for tarsus length included as a covariate; *Χ* ^2^ = 401.57, 1 d.f., *p* < 0.001), we also found significant differences between sexes (males were relatively heavier than females; *Χ* ^2^ = 9.41, 1 d.f., *p* = 0.002) and a significant effect of treatment (*Χ* ^2^ = 55.27, 2 d.f., *p* < 0.001; Figure S2). Chicks from the nitrate-exposed groups (N100 and N500) were significantly heavier than those in the control group, 5.5% and 3.4%, respectively (both *p* < 0.001).

Under stress situations, such as exposure to contaminants, birds would typically be expected to reduce food intake and, consequently, their condition.^77^ Contrary to this expectation, in our experiment, we observed a weight increase during nitrate exposure. Although the underlying mechanisms remain uncertain, several physiological pathways could explain this pattern, potentially resulting from the disruptive effect of nitrates. One potential mechanism is the disruption of hormonal regulation, as nitrate exposure is known to interfere with the thyroid hormone axis in animals.^78–80^ Several studies have reported a decrease in T3 and T4 levels in animals exposed to nitrates,^81,82^ which could lead to a reduction in basal metabolic rate and sodium and fluid retention, ultimately contributing to weight gain.^83,84^ Nitrates may also impair kidney function, leading to fluid retention mediated by hormonal responses such as vasopressin release, which increases body water content.^85,86^ The observed weight gain may also partly result from organ weight increases following nitrate exposure. For instance, Rodríguez-Estival et al.^31^ reported increased spleen weight in birds exposed to the same nitrate concentrations as our study for 14 days, while González Delgado et al.^68^ observed spleen and liver enlargement in nitrate exposed rats. However, organ weight changes alone may not explain the overall weight gain, since, for example, Eskiocak et al.^82^ reported a significant increased thyroid gland weight (together with a reduction in thyroid hormones), while total body weight remained unchanged in rats exposed to sublethal nitrate concentrations (0–500 mg/L over 30 days). Finally, after one month of exposure, we found no treatment effects on growth or condition. The wing or tarsus length of 60-day-old chicks only differed between sexes (*Χ* ^2^ = 73.43, 1 d.f., *p* < 0.001 and *Χ* ^2^ = 200.18, 1 d.f., *p* < 0.001, respectively, males being larger than females). Body condition (weight corrected for tarsus length; *Χ* ^2^ = 162.86, 1 d.f., *p* < 0.001) did not differ between treatment groups or sexes one month after the end of exposure. This indicated that the weight gain in chicks observed during nitrate exposure reversed over time once the exposure ended.

### 3.2 Effects on Antipredator Behavior

Associations between nitrate exposure and behavioral changes, including antipredator responses, have mainly been investigated in aquatic organisms due to their higher risk of exposure.^39,80,87,88^ However, studies investigating these effects of nitrates in birds are limited and focus mainly on feeding and reproductive end points,^32,89^ making this the first study, to our knowledge, to report effects of nitrate exposure on antipredator behavior in birds. We found that the fleeing distance, at which partridges reacted to the predator, was influenced by treatment depending on predator type (Tables S2 and S3). Partridges overall had greater fleeing distance in response to the fox compared to the raptor (*p* < 0.001), indicating they took longer to respond to an aerial attack; conversely, when responding to the fox, partridges reacted from a greater distance (quicker response to terrestrial predators). This aligns with previous literature suggesting that partridges typically exhibit a more active escape to terrestrial predators.^90–92^ We also found an effect of nitrate exposure on fleeing distance, with shorter fleeing distances in the N100 and N500 treatments than in the control group (Figure 2a,b; *p* = 0.008 and *p* = 0.011, respectively), regardless of the type of predator (nonsignificant treatment × predator type interaction). These effects (reduced reaction time to both aerial and terrestrial attacks in exposed birds) could significantly affect the difference between escape success or failure in response to a real predator attack,^93^ implying an increased predation risk for nitrate-exposed chicks.

**Figure 2:**
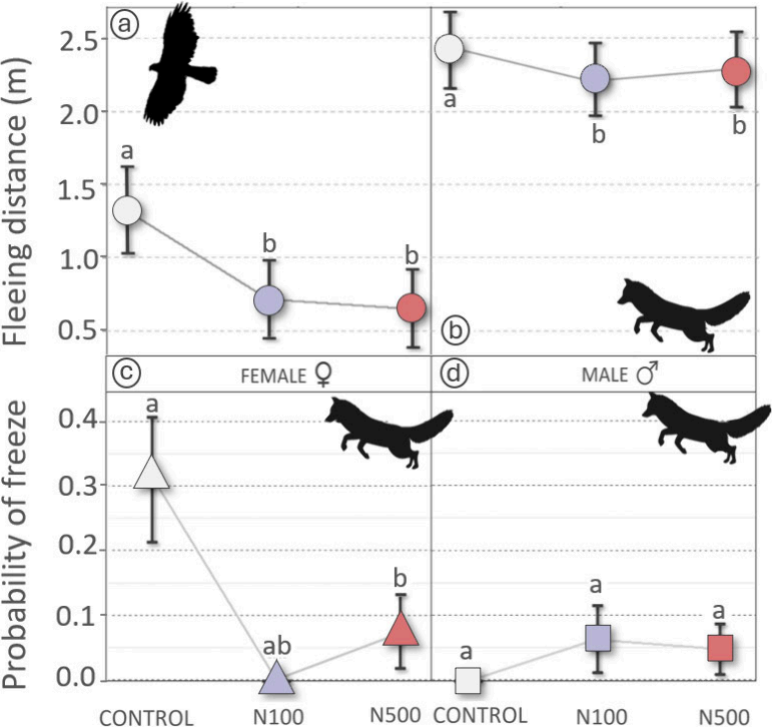
Behavioral responses of exposed partridges to simulated raptor and fox approaches. Top row: Fleeing distances (mean ± SE, in meters), defined as the distance between the predator and the partridge chick when they initiated the escape response, shown by treatment and predator type (a: raptor; b: fox). Bottom row: Freezing probability (mean ± SE), referring to the chick’s immobility or “freezing” behavior in response to the fox, shown by treatment and sex (c: female; d: male). Different letters above the error bars indicate statistically significant differences between groups (*p* < 0.05).

Additionally, the probability of freezing upon detecting a predator was primarily influenced by the chicks’ initial alert state (with calmer individuals more likely to freeze, Tables S2 and S3; *p* < 0.001), but significant interactions were also found between predator type and sex, as well as a three-way interaction involving predator type, treatment, and sex (Table S2). To further explore these interactions and elucidate treatment effects, we conducted separate analyses for freeze behavior in response to each predator (fox and raptor) and then by sex. The exposure to nitrates had no significant effect in response to the raptor, irrespective of sex (Figure S3); it was only influenced by the chick’s initial state of alertness (*Χ* ^2^ = 6.74, 2 d.f., *p* = 0.034), with calmer chicks showing a higher tendency to freeze. Meanwhile, the probability of freezing in response to the fox also varied with the chick’s initial alarm state (as shown in the initial model; *Χ* ^2^ = 5.69, 1 d.f., *p* = 0.017) and was reduced by treatment (*Χ* ^2^ = 12.50, 2 d.f., *p* = 0.002) but depended on sex (sex effect: *Χ* ^2^ = 10.71, 1 d.f., *p* = 0.001; treatment by sex interaction: *Χ* ^2^ = 8.90, 2 d.f., *p* = 0.012). Considering treatment effects by sex, we found significant differences in females based on initial alarm state (Figure 2c; *Χ* ^2^ = 4.59, 1 d.f., *p* = 0.03) and treatment (*Χ* ^2^ = 12.508, 2 d.f., *p* = 0.002). Tukey posthoc tests indicated that freezing probability was marginally higher in control females than in females exposed to the N500 concentration (*p* = 0.1). On the other hand, males consistently exhibited low freezing behavior across all treatments (Figure 2d). The freezing behavior, where the bird remains motionless and crouches, is an effective strategy used by birds in open spaces, as it combines camouflage with reduced movement to avoid detection by predators.^92,94^ In red-legged partridges, this passive behavior is used primarily during aerial attacks,^90^ although no prior sex differences had been reported. Consequently, the reduction in freezing behavior due to agricultural toxicants likely increases the visibility of partridges to predators. Addy-Orduna et al.^41^ reported that exposure to neonicotinoids, insecticides commonly used in seed treatments, altered the freezing and crouching behaviors of partridges; however, no studies have yet examined the effects of nitrates on bird behavior. Studies on amphibians have documented changes in passive behavior, such as predator detection and immobility responses, after exposure to nitrogenous fertilizer ammonium nitrate (NO_3_NH_4_), at concentrations typical of agricultural environments. For example, Ortiz-Santaliestra et al.^95^ found that tadpoles of two toad species exposed to this fertilizer were more susceptible to predation by crayfish, likely due to impaired predator detection. Similarly, Dimitrova et al.^39^ demonstrated that nitrate altered the antipredator motionless behavior of common toads (*Bufo bufo*), equivalent to the freezing behavior in birds. However, comparisons with these studies should be made cautiously, as they involved different taxa and ammonium nitrate exposure, whereas our study used sodium nitrate, making it difficult to determine the specific contribution of each ion to the reduced antipredator performance, particularly given the known neurotoxicity of ammonium.^27^

Regarding alarm calling in the presence of a predator, we only found an influence of testing order (alarm probability was lower during the second test, irrespective of treatment or predator type; nonsignificant interactions; Table S2, Figure S4). However, we observed treatment effects on other active responses to predators (escape distance and refuge use; Tables S1 and S3). Maximum escape distance was significantly greater in the N500 treatment group than in the control group (Figure 3a; *p* = 0.013). Escape distance also differed between sexes and according to predator type (Table S2), with males showing greater distances than females (Figure S5a; *p* < 0.031) and longer escape distances in response to the fox than to the raptor (Figure S4b; *p* < 0.003). Regarding refuge use, we found that chicks exposed to the highest nitrate concentration were less likely to reach the refuge than those from the N100 or control groups, although differences were only marginally significant (Figure 3b; *p* = 0.070). In addition, probability of reaching the refuge was higher in response to the fox than to the raptor (Figure S5c; *p* = 0.031) and during the second test than during the first one (Figure S4d; *p* = 0.001). Alterations in active responses, such as greater maximum escape distances of exposed chicks (N500) and difficulty getting to the refuge, may be a compensatory mechanism for their delayed response described above (shorter fleeing distance at which partridges reacted to the predator). Fear, which is associated with antipredator behavior, induces context-dependent responses,^96,97^ so a delayed detection may lead to a more intense escape response when the predator is closer, causing exposed chicks to run further to avoid capture and to make less use of the refuge. In contrast to our findings, Secondi et al.^98^ reported no effect of nitrate exposure at environmental concentrations (75 mg/L NO_3_) on the escape behavior of palmate newts (*Lissotriton helveticus*) after 10 days of exposure. However, other studies on nitrogenous fertilizers at a negative concentration have shown negative effects. For instance, García-Muñoz et al.^99^ observed impaired escape performance in anuran larvae, while Ortiz-Santaliestra et al.^95^ reported reduced overall activity and altered swimming patterns in *Pelobates cultripes* tadpoles when escaping crayfish predators. Nevertheless, as previously noted, differences in taxa and nitrogen compounds limit direct comparisons with those in our study.

**Figure 3:**
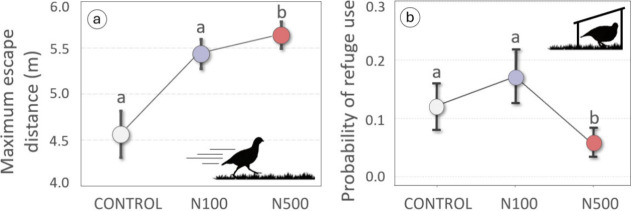
Active responses of exposed partridges to simulated predator approaches. (a) Maximum escape distance (mean ± SE, in meters), defined as the total distance covered by the partridge chick while fleeing from the predator, shown according to treatment. (b) Probability of refuge use (mean ± SE), referring to the likelihood that a chick entered a refuge as part of its escape response, also shown by treatment. Different letters above the error bars indicate statistically significant differences between treatments (*p* < 0.05).

To understand population and ecosystem impacts of contaminants, behavioral end points are increasingly recognized as sensitive indicators for detecting the more subtle effects of environmental toxicants.^41,100^ A single behavioral parameter often provides a more comprehensive assessment than physiological or biochemical measures, as it reflects the integrated outcome of various underlying processes.^101^ Therefore, the behavioral effects observed in this experiment may be linked to molecular and physiological alterations. One key mechanism by which nitrate exposure could alter behavior is through the hypoxic conditions caused by nitrate-induced methemoglobinemia discussed above, as hypoxic conditions have been shown to affect antipredator behavior in fish.^37,102^ Escape responses in many animals are typically anaerobic, as they rely on brief bursts of activity primarily driven by white muscle fibers, suggesting a limited impact from hypoxia on these short-term events.^103,104^ However, to maintain an active escape behavior, red muscle fibers are also involved in birds, which require aerobic metabolism,^105^ and hypoxia could reduce their performance. Moreover, in vivo models of cerebral hypoxia suggest that oxidative damage and ionic homeostasis disruptions can cause neuronal death,^106^ impairing brain and sensory functions that could disrupt neural pathways essential for effective escape responses. As seen before, another potential mechanism is the disruption of the thyroid hormone axis by altering gene expression in animals,^79,107–109^ since thyroid hormones play a crucial role in neurodevelopment and the modulation of behaviors such as locomotion, reproduction, and antipredator responses.^110–112^ In birds, nitrate exposure has been shown to alter circulating concentrations of thyroid hormones (T3 and T4),^81^ and mutations in genes involved in thyroid hormone synthesis have been documented to affect aerial antipredator behavior in birds.^113^ In addition, another explanation for the effects of nitrates on bird behavior could be that, under physiological conditions, gut bacteria reduce nitrate to ammonia via catalytic reduction.^26^ Ammonia affects the nervous system by altering glutamate neurotransmission, directly influencing GABA receptors, and disrupting monoaminergic neurotransmitter systems.^27^ The unionized form of ammonia (NH_3_) can affect vertebrate nervous systems by replacing potassium at nerve membranes,^114^ and at the sarcolemma, this substitution can lead to depolarization of white muscle,^115,116^ impairing the quick escape movements.^103^

The behavioral responses observed following sublethal exposure to nitrates can affect the ability of partridge chicks to escape predators and survive in natural conditions, particularly during summer, when the risk of exposure to nitrates in fertigation water is increased because of a reduced availability of alternative drinking sources. Field studies have revealed that predation is a main driver of population dynamics of red-legged partridges, with mortality attributed to terrestrial predators being higher than that attributed to raptors.^75,93,117^ Additionally, the observed effect on antipredation behavior could be further exacerbated in natural environments through other alterations reported in this study, such as changes in body condition, increased hematocrit, and a weakened immune system, factors that could increase the costs of escape behaviors, making birds more susceptible to predation. However, extrapolating the behavioral effects observed in our study to field conditions may be limited. Active antipredator behaviors are often learned, unlike freezing, which has both genetic and learned components.^118–120^ While the observed responses align with previous studies on red-legged partridges,^121–123^ our captive-reared partridges lacked exposure to adult models during rearing, limiting their opportunity to learn exemplary behaviors.^117^

### 3.3 Implications for Environmental Risk Assessment

The Environmental Protection Agency (EPA) sets the safe nitrate level for drinking water at 10 mg/L as nitrate-nitrogen, equivalent to 44.3 mg/L nitrate, and The World Health Organization (WHO) reports that acute oral toxicity of nitrates, as indicated by median lethal dose (LD50) studies, ranges from low to moderate in most mammals (LD50 1600–9000 mg/kg).^124^ In contrast, birds appear to be more sensitive to nitrate toxicity; for instance, studies show an LD50 of 619 mg/kg in northern bobwhite (*Colinus virginianus*)^125^ and an LD50 of 659 mg/kg in turkey vultures (*Cathartes aura*).^126^ Regarding the long-term toxicity of nitrate thresholds, there is a lack of information specifically related to birds. In our study, the 28-day exposure resulted in estimated average daily dietary doses of 15.5 mg/kg bw/day for the low treatment and 75.2 mg/kg bw/day for the high treatment, with observable effects at both levels. This suggests that the Lowest Observed Adverse Effect Level (LOAEL) for nitrate in birds may be below 15.5 mg/kg of b.w./day. However, specific toxicity studies to determine toxicological thresholds, such as LD50, LOAEL, or No Observed Adverse Effect Level (NOAEL) values, should be properly conducted for susceptible species to effectively incorporate them into environmental risk assessments.

Our study demonstrates that long-term nitrate exposure can significantly affect the physiology and behavior of birds, with the simulated scenarios reflecting conditions likely to occur in the field. GPS tracking has shown an increased use of vineyards by wild partridges during the summer, where nitrate concentrations in fertigation water were like those in our study (100–500 mg/L), although in some cases, concentrations could reach up to 1100 mg/L (authors, unpublished data). This concentration could result in nitrate doses of up to 110 mg/kg of b.w. in a single day for an adult partridge weighing 400 g based on water consumption data from our experiment (approximately 10% of body weight), representing an extreme exposure scenario. Limited water sources and high fertigation use during the dry season^42,127^ could increase nitrate exposure risk for birds, with dehydration, a reported effect, that could exacerbate water consumption and create an ecological trap that may compromise individual survival.^128^ The effects on growth, immunity, and survival of exposed chicks could translate into long-term consequences, as an increase in chick mortality could be contributing to the red-legged partridge population declines reported in recent years in Spain,^45,46^ raising serious concerns about the risk posed by nitrates to wild bird populations.

Experimental studies like ours, combined with investigations of potential exposure in real field scenarios, reveal a significant gap in the European risk assessment of nitrate use in agricultural contexts and its impacts on wildlife.^129,130^ In fact, experts have highlighted the absence of a comprehensive methodology for evaluating the environmental risks associated with fertilizer use in agricultural areas.^129–132^ The main regulatory framework for fertilizers in Europe is Registration, Evaluation, Authorization, and Restriction of Chemicals (REACH) (Regulation (EC) No. 1907/2006), which primarily focuses on human health and environmental protection but does not directly evaluate specific effects on wildlife. Additionally, the EU Nitrates Directive (Directive 91/676/EEC) regulates nitrate use in agriculture to protect vulnerable zones but mainly targets groundwater contamination from nitrate leaching of agricultural origin. Although there is guidance for environmental exposure assessment of fertilizers under REACH regulation (e.g., ECHA, 2020 ^133^), this approach relies on Predicted Environmental Concentration (PEC) calculations and risk characterization ratios (RCRs) for nitrates to assess potential toxicity risk in nontarget organisms. This approach to risk assessment may be insufficient due to its generalized nature, relying on hydrological and chemical models of nitrate behavior that often overlook critical exposure scenarios, such as when birds or other wildlife ingest fertigation water from small puddles, which can significantly exceed the EPA’s safe limit equivalent to 44.3 mg/L nitrate. Our findings highlight the need for more specific risk assessments of nitrates used in agricultural fertigation, incorporating realistic exposure scenarios and toxicological effects at these concentrations to help understand and mitigate the impact of these practices on farmland birds, whose populations are in decline.

Our results show that nitrate exposure through irrigation water, at concentrations commonly used in fertigation practices, can negatively affect multiple levels of biological organization in red-legged partridge chicks. We observed physiological alterations, such as increased hematocrit and suppressed cell-mediated immune responses, along with behavioral changes that impair effective antipredator responses. Notably, this is, to the best of our knowledge, the first time that these behavioral effects have been documented in birds exposed to nitrates under a realistic exposure scenario. These chronic sublethal effects may directly impact bird fitness and population viability in intensively farmed areas, where fertigation is widely applied. In this context, our findings support the implementation of mitigation measures, such as providing access to uncontaminated water sources, particularly during the summer months, when water is scarce and chick development overlaps with peak irrigation. Finally, we recommend that current regulatory frameworks consider incorporating specific risk assessments for nitrogen-based fertilizers under high-exposure scenarios, such as fertigation, with particular attention to sublethal and behavioral end points in vulnerable wildlife species.

## Supplementary Material



## Notes

Statistical analysis details; results of treatment effects on cell-mediated immune response, hematocrit, wing and tarsus length, and body weight in red-legged partridge chicks; detailed GLM output testing effects of nitrate exposure on behavioral responses to simulated predator approaches; results of active (alarm calling, escape distance, shelter use) and passive (predator detection, freezing behavior) antipredator behaviors; schematic representation of the predator simulation test; figures showing treatment- and sex-specific effects on chick condition; freezing probability by sex and treatment; alarm calling probability by test order; maximum escape distance (mean ± SE) by sex and predator type; predicted shelter use probability (mean ± SE) by sex and test order (PDF)
