# Supplementary material for: Effect of Fertigated Water Consumption on the Immune Responsiveness and Antipredator Behavior of Red-Legged Partridge Chicks
Source: Environ Sci Technol. 2025 Jun 24;59(26):13169–80. doi: 10.1021/acs.est.5c01484 (PMC12243088; doi:10.1021/acs.est.5c01484)
Supplement: Supplementary file 1 [file es5c01484_si_001.pdf]

SUPPLEMENTARY INFORMATION

Effect of fertigated water consumption on the immune responsiveness and antipredator behavior of red-legged partridge chicks

Elena Fernández-Vizcaíno[1], Mario Fernández-Tizón[1], Rocío Tarjuelo[1,2], Manuel E. Ortiz-Santaliestra[1], Rafael Mateo[1,3] & François Mougeot[1]

[1] *Instituto de Investigación en Recursos Cinegéticos (IREC) CSIC-UCLM-JCCM,13005, Ciudad Real. Spain.*

[2] *Dpto. Biogeografía y Cambio Global, Museo Nacional de Ciencias Naturales (CSIC), Spain.*

[3] *Institute for Environmental Assessment and Water Research (IDAEA-CSIC), Jordi Girona 18, 08034, Barcelona, Spain.*

*E-mail contact: efvval@hotmail.com

Number of pages: 9

Number of tables: 3

Number of figures: 5

1. MATERIAL AND METHODS

1.1 Statistical analysis

To evaluate whether water consumption was affected by treatment, we used a Generalized Linear Model (GLM) with the estimated water consumption per partridge as the dependent variable and treatment, date and the interaction between these two variables as explanatory variable. To determine if nitrate consumption influenced the growth of chicks, we tested for differences in tarsus and wing length at two ages: when chicks were 35 days old (end of the exposure period) and one month later (60 days old chicks). We conducted two GLMs (one for each age), with wing length or tarsus length as the dependent variables and treatment, sex, and their interaction as fixed factors. Similarly, we tested for differences in chick condition (weight corrected for size) at age 35 and 60, by modelling variation in body weight (g) and adding tarsus length as a covariate in our model (as an index of structural size). We added the explanatory variables treatment, sex, and their interaction to test for treatment effects.

To assess whether immunity and hematocrit differed between treatment groups, we conducted three GLMs. The dependent variables in these models were the cell-mediated immunity (CMI) response or hematocrit percentage. The explanatory variables were treatment, sex, and their interaction. We included body condition as a covariate in the analysis of CMI responsiveness as CMI is condition-dependent in red-legged partridges.

To investigate nitrate effects on the antipredator response of chicks, we used a GLM with a binomial error distribution and a logit link function. The dependent variable was the freezing probability in response to the predator, and the factors included test order (fox first vs raptor first), the chick's initial state, predator type (fox or raptor), treatment, sex, and the interactions between these factors, including a three-way interaction between predator, sex, and treatment. Since this three-way interaction was significant, and to further explore differences between groups, we ran two separate GLMs, one for each predator (fox or raptor), with the following factors: test order (first versus second test), the chick's initial state, treatment, sex, and the interactions between treatment and sex.

To determine which factors influenced fleeing distance (the distance at which the partridge reacted to the predator's approach) and the maximum escape distances in response to the predator, we conducted GLMs using the fleeing or maximum distance of birds that did not exhibit freeze behavior as the dependent variable. The test order, the chick's initial state, predator type, treatment, sex, and the interactions between these factors, including a three-way interaction between predator, sex, and treatment, were included as explanatory variables.

To investigate the factors that may affect the alarm calling by partridges in response to a predator, we ran a GLM with a binomial error distribution and a logit link function. The dependent variable was the alarm calling probability, and the factors included test order (first vs. second test), the chick's initial state, predator type (fox or raptor), treatment, sex, and their interactions, including a three-way interaction between predator, sex, and treatment. Finally, to investigate the factors that may affect the probability of reaching the shelter, we ran a GLM with a binomial error distribution and a logit link function and the following explanatory variables: test order (first vs. second test), the chick's initial state, predator type (fox or raptor), treatment, sex, and their interactions, including a three-way interaction between predator, sex, and treatment.

Initially, the models included all factors, covariates, and their interactions. Non-significant terms were sequentially removed using a backward selection procedure, starting with interactions. The treatment factor was retained in the models to report on the (lack of) significance of treatment effects, and Tukey tests were used to test for pairwise differences between the three treatment groups when significant differences were dected. For all tests, the significance level was set at $p < 0.05$, considering p-values between 0.05 and 0.1 as marginally significant.

2. TABLES

Table S1. Mean values (±SD) of cell mediated immune response, hematocrit, wing and tarsus length and body weight in chick red-legged partridges according to treatment. Values in bold with asterisks (*) indicate expression levels different from those of controls at the $p < 0.05$.

	CONTROL			N100			N500		
	N	Mean	SD	N	Mean	SD	N	Mean	SD
Consumption of water/partridges/day (L)	5	0.022	0.001	5	0.024	0.001	5	0.023	
Cell mediated inmune response	32	63.5	26.6	33	**48.6***	22.0	35	**43.7***	30.2
Hematocrit (%)	17	29.0	2.0	17	**32.0***	2.0	24	**33.0***	2.0
Weight at the end of exposure (g)	32	147.3	20.1	35	**155.4***	17.9	34	**152.8***	19.2
Wing length at the end of exposure (cm)	32	122.6	5.9	35	123.4	5.6	34	121.3	5.9
Tarsus length at the end of exposure (cm)	32	42.6	2.2	35	42.0	1.9	34	41.8	2.2
Weight 1 month after exposure (g)	30	297.2	29.3	35	304.3	29.5	34	300.1	31.4
Wing 1 month after exposure (cm)	30	149.9	5.3	35	150.1	5.8	34	149.6	6.4
Tarsus 1 month after exposure (cm)	30	51.2	2.0	35	51.2	1.9	34	51.1	2.1

Table S2. Results of the final GLMs (X^2, d.f., and p-value) testing for effects of nitrate exposure on the chicks' behavioral responses to simulated predator approaches (freezing probability, alarm calling probability, fleeing distance, maximum escape distance, and shelter use). The left column includes all factors and interactions used in the initial models. Non-significant factors and interactions were sequentially removed following a backwards selection and the table shows only those retained in the final models.

	Freezing probability			Alarm calling probability			Fleeing distance			Maximum escape distance			Shelter use		
	X^2	df	p	X^2	df	p	X^2	df	p	X^2	df	p	X^2	df	p
Sex	0.019	1	0.891							4.76	1	0.029			
Order				9.26	1	0,002							13.87	1	0.000
Estate	11.44	1	0.001												
Predator	1.07	1	0.300				163.39	1	0.000	9.00	1	0.003	3.16	1	0.076
Treatment	0.37	2	0.829	0.62	2	0.732	11.37	2	0.003	8.32	2	0.016	5.51	2	0.064
Order*Predator															
Predator*Treatment	5.26	2	0.072												
Sex*Treatment	4.99	2	0.082												
Sex*Predator	6.90	1	0.009												
Sex*Predator*Treatment	9.87	2	0.007												

Table S3. Mean ± SD, minimum, and maximum values of flight initiation distance and maximum escape distance in red-legged partridge chicks and the percentage of chicks per treatment displaying freezing, alarm and refuge use behaviors categorized by treatment and predator.

		Control					N100					N500				
		N	Mean	SD	Max	Min	N	Mean	SD	Max	Min	N	Mean	SD	Max	Min
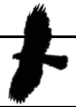	Fleing distance	33	0.91	1.01	3.50	0.00	39	0.60	0.61	2.50	0.00	36	0.68	0.61	2.00	0.00
	Max. Escape distance	33	4.42	1.95	6.00	1.00	39	4.97	1.78	6.00	1.00	37	5.54	1.37	6.00	1.00
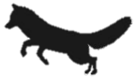	Fleing distance	33	1.93	1.20	3.80	0.00	39	2.23	0.76	3.80	0.00	37	2.14	0.91	3.20	0.00
	Max. Escape distance	33	4.70	2.17	6.00	1.00	40	5.88	0.79	6.00	1.00	37	5.73	1.15	6.00	1.00

		Control		N100		N500	
		N	% of positive	N	% of positive	N	% of positive
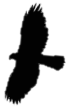	Freeze	33	21.21	39	12.82	37	5.41
	Alarm	33	27.27	39	23.08	37	29.73
	Refuge use	33	6.06	39	20.51	37	5.41*
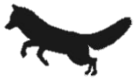	Freeze	32	18.75	40	2.50	37	5.41
	Alarm	32	15.62	40	25.00	37	24.32
	Refuge use	33	24.24	40	20.00	37	13.51

3. FIGURES

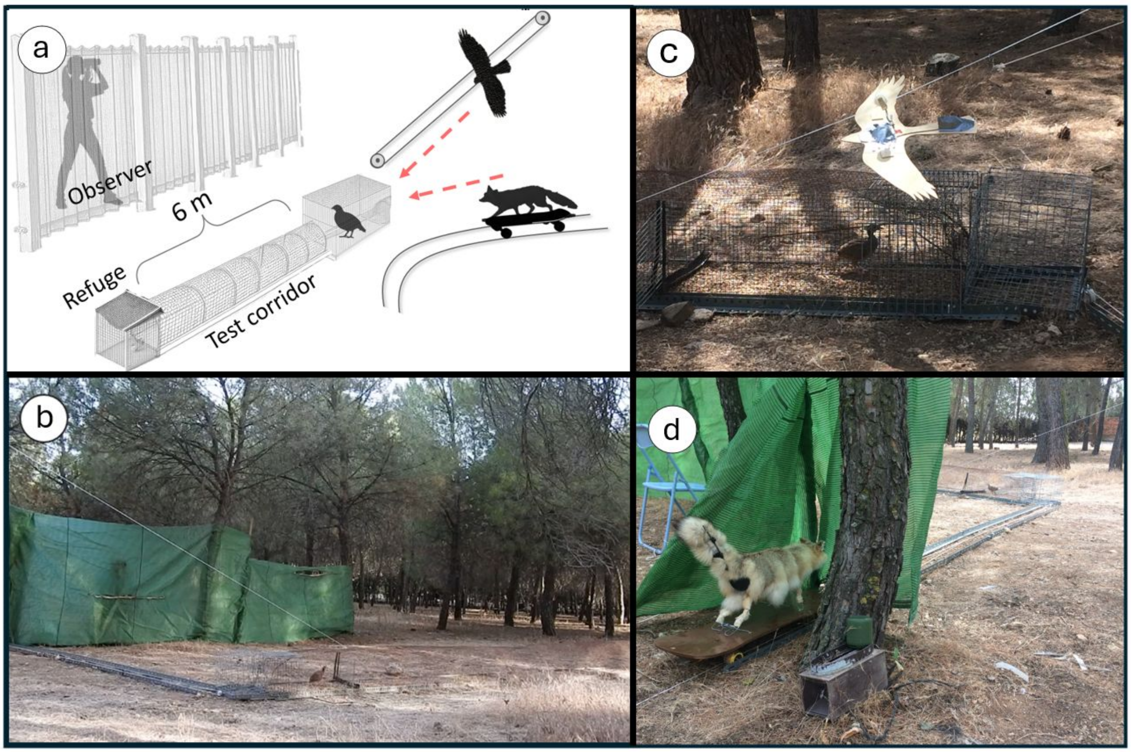

Figure S1. a) Schematic representation of the simulated predator approach tests b) Photograph of the outdoor predator testing system; c) Photograph of the raptor approach simulation; d) Photograph of the fox approach simulation.

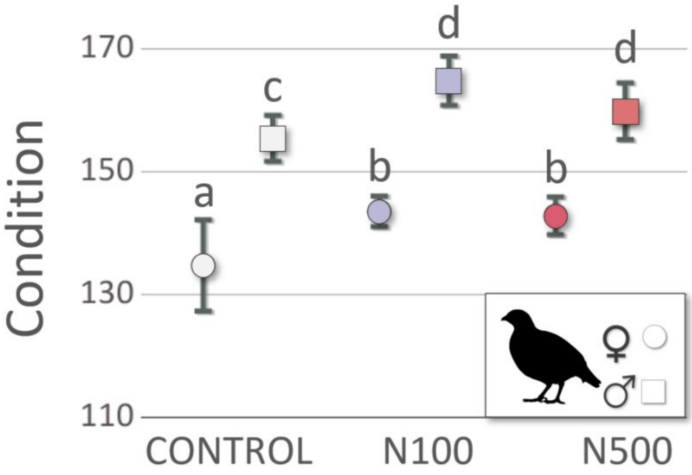

Figure S2. Mean (±SE) condition (body weight relative to tarsus length) of partridge chicks according to treatment group (colors) and sex (symbols). Different letters above error bars indicate significant differences between groups at the $p < 0.05$ level.

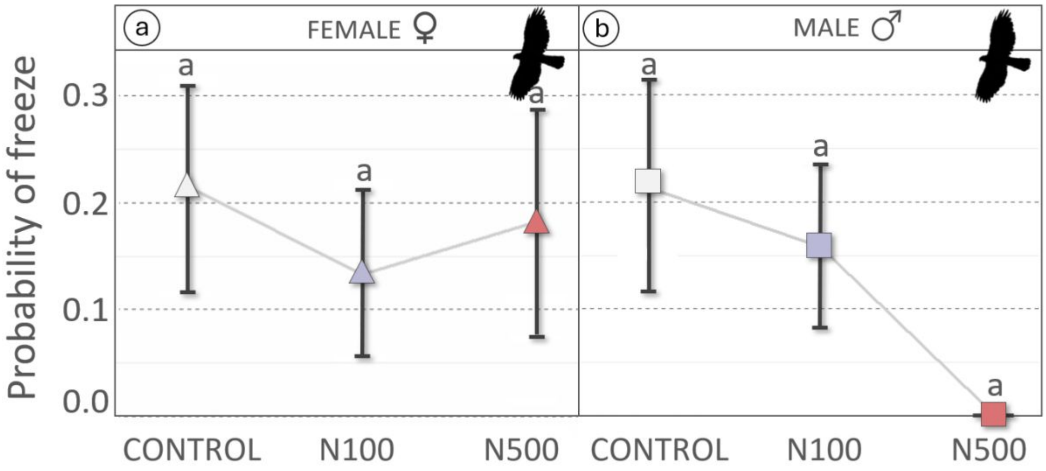

Figure S3. Freezing probability (±SE) of partridges exposed to a raptor, according to sex and treatment.

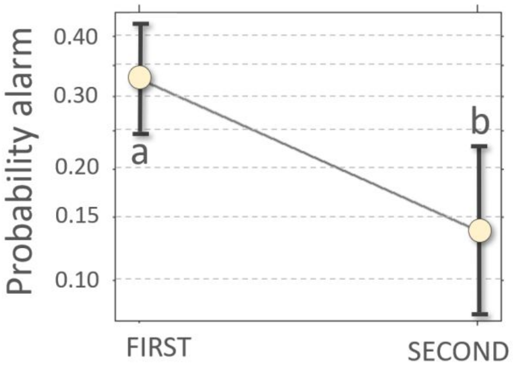

Figure S4. Alarm calling probability (±SE) of partridges according to test order. Different letters above or below the error bars indicate significant differences between groups at the $p < 0.05$ level.

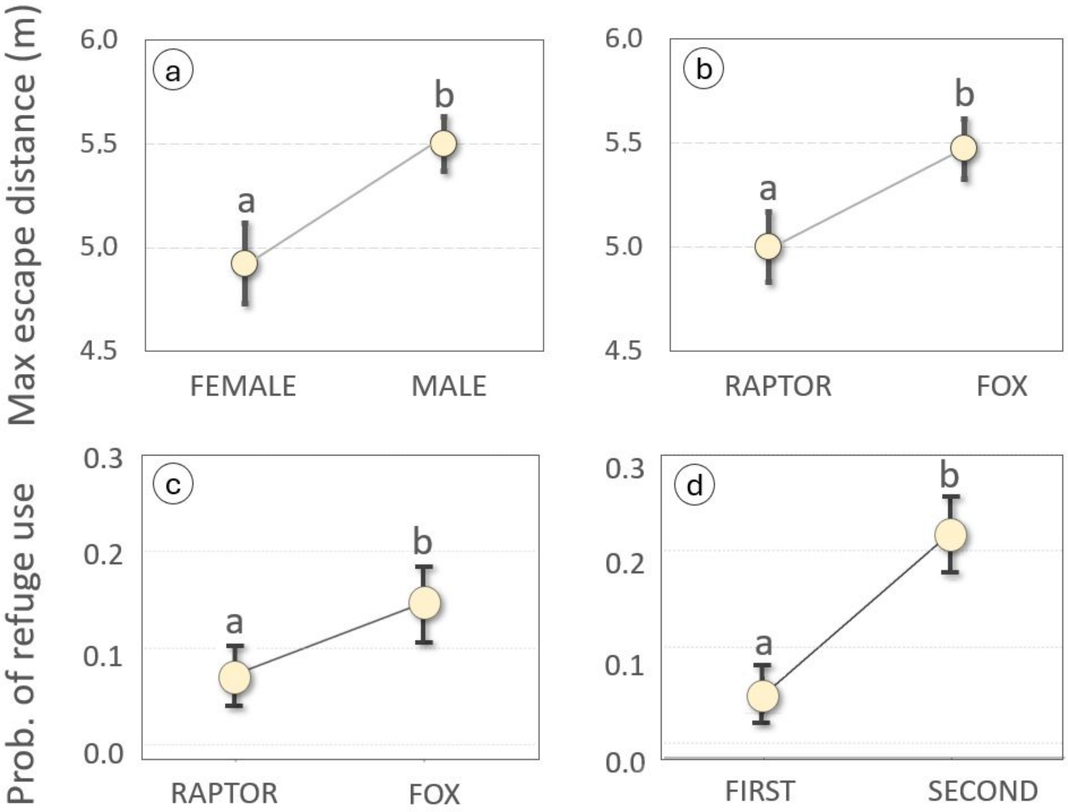

Figure S5. Maximum escape distance (mean± SE) of partridge chicks in response to simulated predator attacks according to sex (a) and predator type (b). Predicted probability of shelter use (mean ± SE) of exposed partridges by sex (c) and test order (d). Different letters above the error bars indicate significant differences between groups at the $p < 0.05$ level.
